# Multiple functions of the scaffold protein Discs large 5 in the control of growth, cell polarity and cell adhesion in *Drosophila melanogaster*

**DOI:** 10.1186/s12861-020-00218-0

**Published:** 2020-06-18

**Authors:** Parvathy Venugopal, Hugo Veyssière, Jean-Louis Couderc, Graziella Richard, Caroline Vachias, Vincent Mirouse

**Affiliations:** 1 iGReD (Institute of Genetics, Reproduction and Development), Université Clermont Auvergne, UMR CNRS 6293 - INSERM U1103, Faculté de Médecine, 28 Place Henri-Dunant, 63000 Clermont-Ferrand, France; 2 present address : School of Biotechnology, Amrita Vishwa Vidyapeetham, Kollam, Kerala 690525 India; 3 present address : University Clermont Auvergne, INSERM U1240, Centre de Lutte Contre le Cancer Jean PERRIN, 58 rue Montalembert, 63011 Clermont-Ferrand, France

**Keywords:** Drosophila, MAGUK, Yorkie, Hippo, Myc, Polarity, Adhesion, Growth

## Abstract

**Background:**

Scaffold proteins support a variety of key processes during animal development. Mutant mouse for the MAGUK protein Discs large 5 (Dlg5) presents a general growth impairment and moderate morphogenetic defects.

**Results:**

Here, we generated null mutants for Drosophila Dlg5 and show that it owns similar functions in growth and epithelial architecture. Dlg5 is required for growth at a cell autonomous level in several tissues and at the organism level, affecting cell size and proliferation. Our results are consistent with Dlg5 modulating hippo pathway in the wing disc, including the impact on cell size, a defect that is reproduced by the loss of yorkie. However, other observations indicate that Dlg5 regulates growth by at least another way that may involve Myc protein but nor PI3K neither TOR pathways. Moreover, epithelia cells mutant for Dlg5 also show a reduction of apical domain determinants, though not sufficient to induce a complete loss of cell polarity. Dlg5 is also essential, in the same cells, for the presence at Adherens junctions of N-Cadherin, but not E-Cadherin. Genetic analyses indicate that junction and polarity defects are independent.

**Conclusions:**

Together our data show that Dlg5 own several conserved functions that are independent of each other in regulating growth, cell polarity and cell adhesion. Moreover, they reveal a differential regulation of E-cadherin and N-cadherin apical localization.

## Background

The accurate development of an organ or an organism requires a robust coordination size and shape control, both at the cell and tissue scales. Among the protein classes involved in such processes, many of them are scaffold proteins. These proteins are devoid of catalytic activity but contain multiple domains of protein-protein interaction [1]. They allow the formation of complexes that are determinant, for instance, for cell polarity, cell adhesion or that are used as a platform for various signaling events.

Membrane-associated guanylate kinase (MAGUK) proteins are typical examples of scaffold proteins [2]. MAGUK domain is a structural unit formed by SH3 domain next to a non-catalytically active guanylate kinase domain. These domains are usually flanked by one or several PDZ domains and potentially other protein-protein interaction domains. Some MAGUK domains recognize phosphopeptides, whereas some others work in cooperation with the adjacent PDZ domain, reinforcing the affinity of the latter for a specific partner [3, 4]. MAGUK proteins also emerge as important modulators of phase separation in cells [5, 6].

Discs Large (Dlg) is a MAGUK protein that was identified in *Drosophila* for its function in epithelial polarity as a determinant of the lateral domain and the neoplastic effect of its mutation [7–9]. Four paralogs of fly Dlg, Dlg1 to Dlg4, are found in mammals. A more divergent member of the family, Dlg5, is also found in fly and mammals with a conserved architecture: a coiled-coil domain, 4 PDZ domains and a MAGUK domain. Dlg5 studies in mammals emphasized a function in epithelial morphogenesis, the knock-out mouse showing mild defects of adherens junction and epithelial polarity in the kidney, the lung and the brain [10, 11]. Dlg5 is also required for N-Cadherin (N-Cad) delivery to the membrane during synaptogenesis [12]. A report in *Drosophila* using partial loss of function conditions in follicle cells also described moderate defect in the recruitment of apical determinants and junctional proteins [13]. This report suggested that Dlg5 acts mainly by a regulation of the apical determinant crumbs (crb). However, it is unclear whether the effect on polarity determinants and adherens junction are causally linked our whether they reflect independent functions of Dlg5 protein. *Drosophila* Dlg5 is also required for the proper collective cell migration of the border cells [14, 15]. Beside these morphogenetic defects, new born *Dlg5* mice are considerably smaller than their wild-type littermates, suggesting an involvement in growth control [10]. Interestingly, Dlg5 has been functionally linked to the hippo pathway both in mammals and in flies, where it interacts and regulates negatively the MAST/hippo kinase [16]. However, whether such a hippo regulation could account for all the growth defects associated with the loss of Dlg5 is not known. Morever, Dlg5 was also identified as a positive regulator of the Target of Rapamycin complex 1 (TORC1) pathway in an in vitro RNAi screen [17].

Here, we identified *Drosophila Dlg5* in an RNAi screen for genes linked to follicular epithelium development and we generated null mutants. These mutants allowed us to show that this gene is involved in the control of growth, both at the cellular and systemic levels. Our results suggest that Dlg5 regulates growth by at least two independent mechanisms. We also confirmed a moderate epithelial polarity defect and show a very strong and specific effect on N-Cad expression whereas E-Cadherin (E-Cad) is not affected. Importantly, we show that polarity defects and Adherens junction defects reflect independent functions of Dlg5.

## Results

### The loss of Dlg5 alters cell autonomously follicle cell growth

**Fig. 1:**
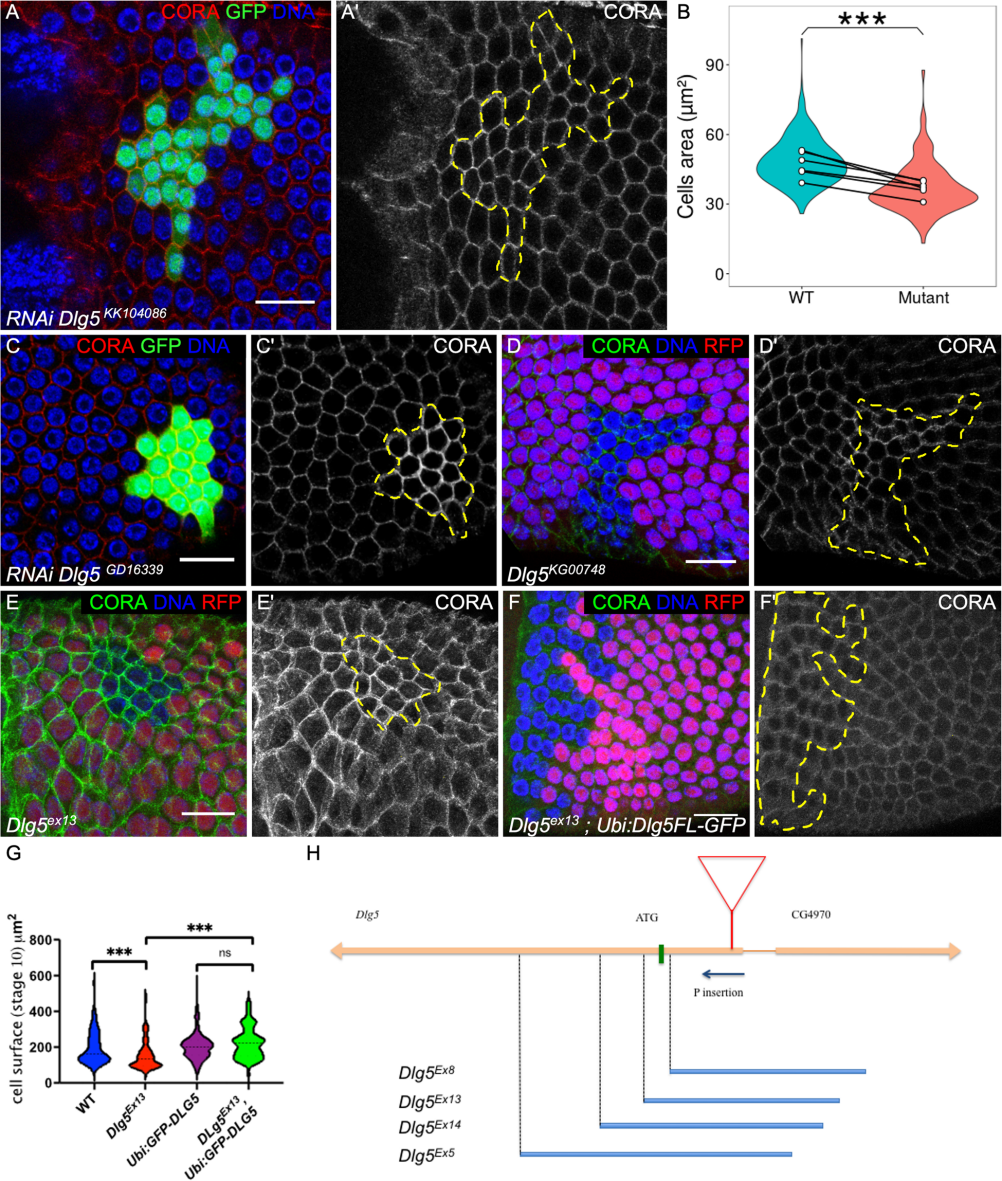
Dlg5 is required for follicle cell growth. **a** and **c** follicle cell clones marked by the GFP and expressing RNAi against *Dlg5* using **a***KK10486* and **c***GD16339* lines and stained with Cora (red and **a**’ and **c**′). **b** violin plot of the quantification of follicle cell size expressing *Dlg5*^*KK10486*^ RNAi clones (mutant) compared to wildtype (WT) surrounding cells on 6 stage 9 follicles. Mean values (white dots) are paired for each follicle. (*P*-value *** < 0,001.) **d**,**e**,**f** mutant follicle cell clone, marked by the absence of RFP, of D) *Dlg5*^*KG00748*^, **e***Dlg5*^*Ex13*^ and **f***Dlg5*^*EX13*^ rescued by a Ubi:Dlg5-GFP transgene. **g** violin plot of the quantification of follicle cell size of the indicated genotypes (*n* > 60 cells from at least 5 independent clones) **h** scheme of Dlg5 locus with the position of the P element KG00748 (red triangle) and the different imprecise excisions that we obtained (blue bars). For all pictures scale bar is 10 μm

We performed a reverse genetics screen to identify new genes involved in *Drosophila* follicular epithelium development, a tissue used as a generic model for various aspects of epithelium biology [18, 19]. Follicle cells form a monolayer epithelium surrounding germline cyst with the apical domain facing the germline. Follicle undergoes a rapid growth through 14 developmental stages, with a 1000-fold volume increase. Follicle cell growth is associated with proliferation until stage 6, then follicle cells become endoreplicative and larger. During the screen, we noticed that clones expressing RNAi against *Dlg5* were small and the cells appeared also smaller than wild-type cells, especially after stage 6 (Fig. 1a). This defect was quantified at stages 9-10A, showing an average reduction of 33% of the cell surface (Fig. 1b). A similar defect was observed with a different RNAi line (Fig. 1c). A P-element insertion in the 5’UTR of *Dlg5* was available. This insertion was lethal and homozygous mitotic clones for this mutant also give small follicular cells (Fig. 1d). However, the defect obtained with this mutant appeared more variable than with the RNAi lines, suggesting that it may correspond to a hypomorphic mutant. We generated P-element excisions and most of them restored the viability of the stock indicating that its lethality was associated with this insertion in *Dlg5* gene. We also obtained several lethal imprecise excisions, *Dlg5*^*Ex5,Ex8,Ex13,Ex14*^ all, except Ex8, deleting the start codon and part of the coding sequence (Fig. 1h). However, they also deleted part of the neighboring annotated gene (CG4970). This gene is only expressed in testis and is very poorly conserved, with no known domains and no ortholog in other insect species. Trans-heterozygous between a Minos element inserted in the coding sequence of this gene (Mimic^MI02472^) and the deletions that we generated complement perfectly in terms of viability and fertility, indicating no essential function of *CG4970*. We also obtained rescue of *Dlg5*^*ex13*^ mutation lethality using a transgene with *Dlg5* coding sequence under ubiquitin promoter and fused to GFP added in N-terminal (Ubi:GFP-Dlg5). Thus, we assumed that *Dlg5*^*ex13*^ could be considered as a bona fide null mutant*.* Mitotic clones for this allele contain cells with a reduced size (Fig. 1e,g), a defect also rescued by Ubi:GFP-Dlg5 (Fig. 1f,g), confirming the cell autonomous role of *Dlg5* in follicle cell growth.

### Dlg5 has a general function in growth

**Fig. 2:**
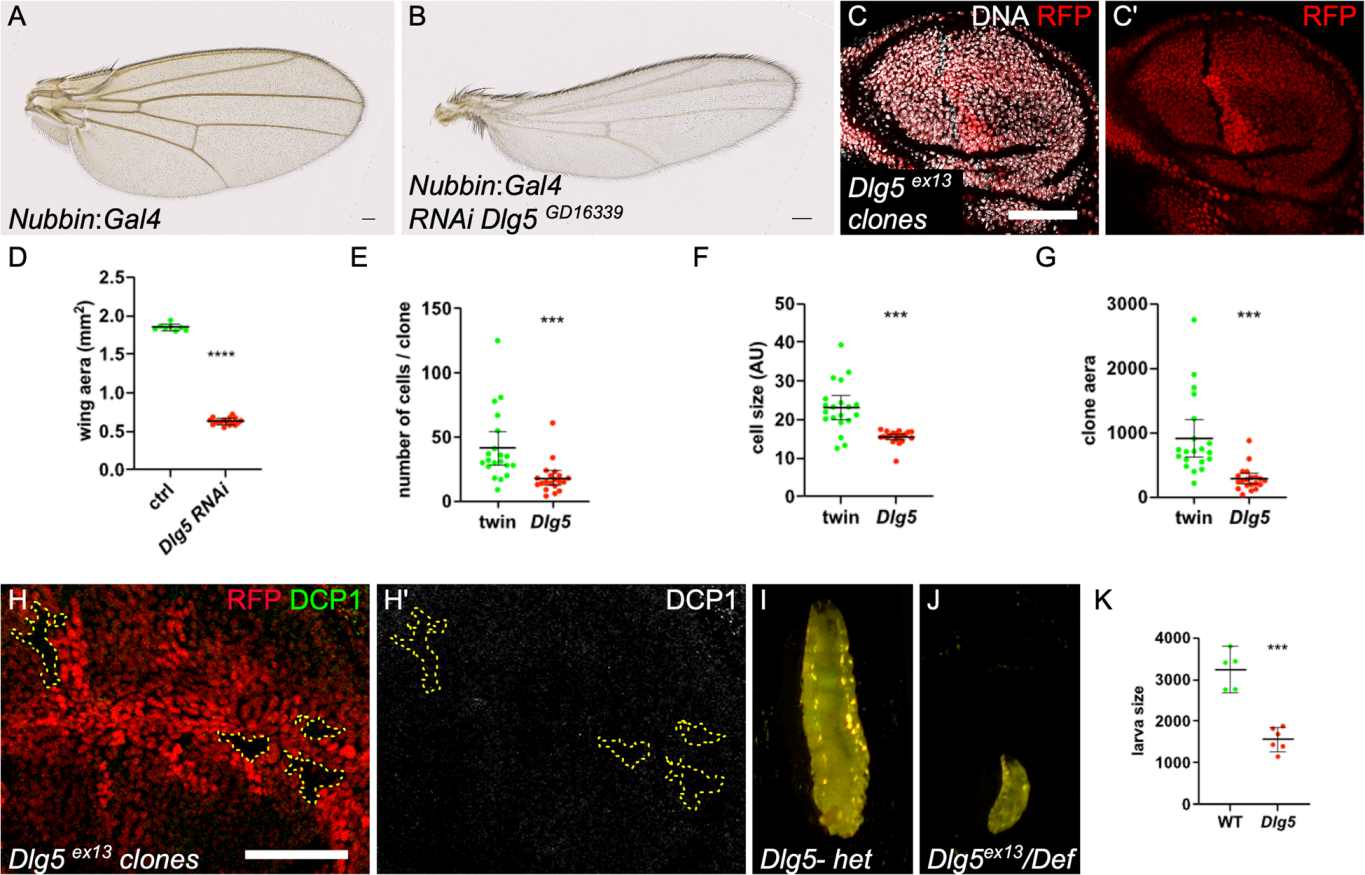
General requirement of Dlg5 for growth. **a** control wing and **b***Dlg5*^*KK10486*^ RNAi expressing wing with Nubbin:Gal4. The area of these wings is quantified in (**d**). **c***Dlg5*^*Ex13*^ mutant clone in the wing disc, marked by the absence of RFP, is smaller than the wild-type twin clone. Mutant clone has an elongated shape. Scale bar 50 μm. **e** cell number **f** mean cell size and **g** total size quantification in *Dlg5*^*Ex13*^ and twin clones in the wing disc of third instar larvae (*n* = 20 for each genotype). **h** and **h′** DCP1 staining does not reveal apoptosis in *Dlg5*^*Ex13*^ mutant clones (outlined in yellow) **i** control (*Dlg5* heterozygous) third instar larva and **j***Dlg5* mutant larva of the same age at 25 °C. **k** quantification of larva size (*n* > 4, *** *p*-value < 0.001)

We next wondered whether Dlg5 function in cell growth could apply to other tissues. We induced Dlg5 knock-down specifically in the wing disc pouch, which corresponds to the future cells of the fly wing, using Nubbin:Gal4 [20]. It led to a dramatic reduction of the wing size (Fig. 2a-b, d). We also induced mitotic clones for *Dlg5*^*Ex13*^ in the wing disc and quantified several parameters. Of notice, the mutant cells tend to form a row of cells rather to extend the clone in all directions, a defect reminiscent of what has been recently described for other mutants generating small cells [21] (Fig. 2c). Mutant clones were systematically smaller than their twin and contained fewer cells (Fig. 2 E,G). Moreover, the mutant cells had, in average, a size reduced by 40% (Fig. 2f). DCP-1 staining did not reveal apoptotic cells in *Dlg5* mutant clones suggesting that the lower cell number per clone correspond to a growth and proliferation decrease (Fig. 2h).

Finally, we looked at a transheterozygous combination of *Dlg5* null alleles. First instar larvae hatch, are able to crawl around and are still alive 48 h after egg deposition. However, their growth is strongly impaired, indicating a systemic requirement for *Dlg5* (Fig. 2j-k). Thus, altogether these results show that Dlg5 owns a general growth function, as its mammal counterpart, and that this function is performed in a cell-autonomous manner.

**Fig. 3:**
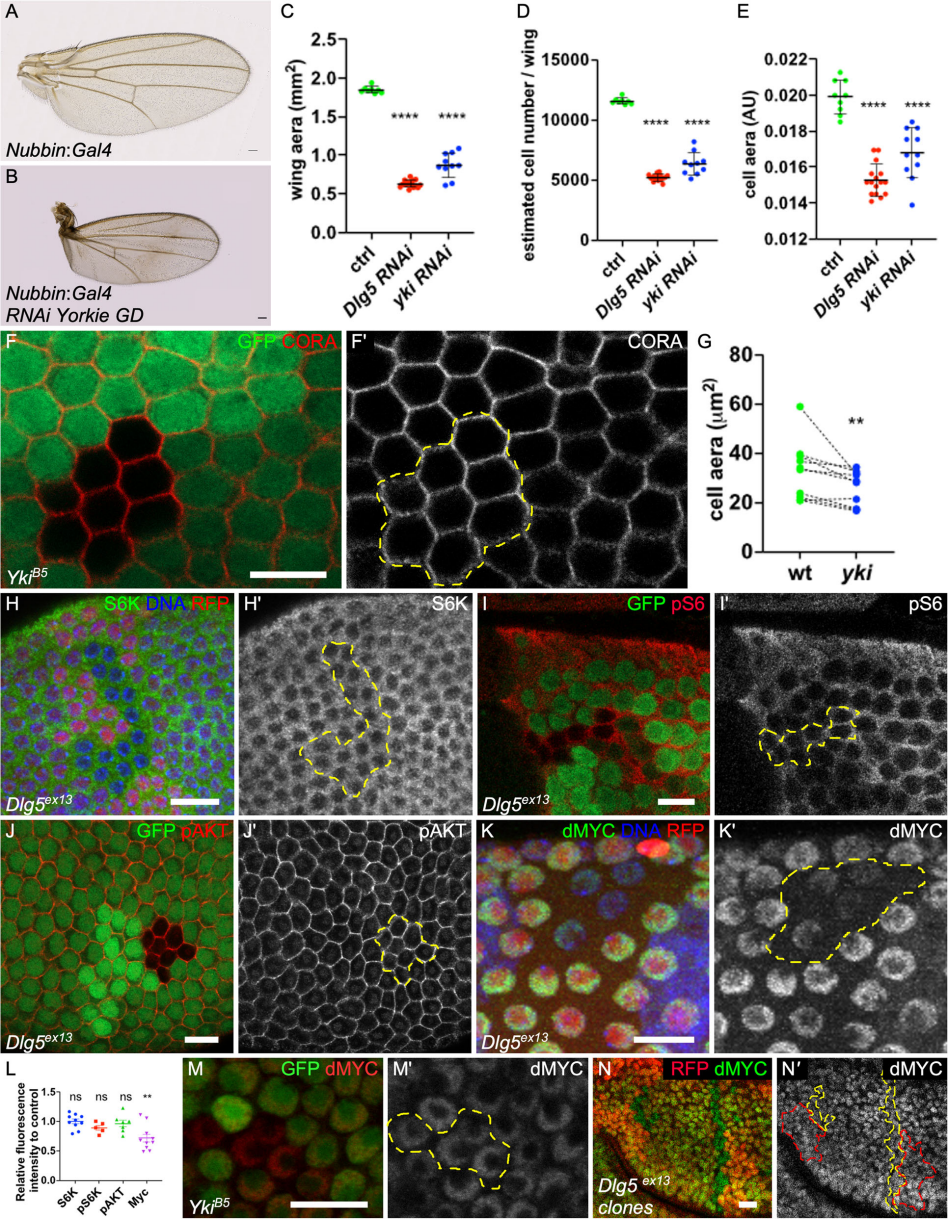
Hippo modulation is not sufficient to explain Dlg5 impact on growth. **a** control wing and **b***yki*^*GD40497*^ RNAi expressing wing with Nubbin:Gal4. **c**, **d**, **e**) quantification of **c** wing aera) **d** cell number and **e** cell size for the indicated genotypes (control *n* = 9, *Dlg5 RNAi n* = 16, *yki RNAi n* = 11). **f** mutant follicle cell clone for *yki*^*B5*^, marked by the absence of GFP at stage 9 and stained for Cora. **g** Quantification of follicle cell size in *yki* clones compared to wildtype (WT) or *yki* heterozygous surrounding cells on stage 9 follicles (*n* = 10). **h-k**) staining for (**h**) S6K, **i** phospho-S6 (pS6) **j** Phospho-AKT (pAKT) and **k** Myc in *Dlg5*^*ex13*^ mutant clones in follicle. **l** Relative quantification of fluorescence for the indicated markers in *Dlg5*^*ex13*^ clones compared to WT surroundings cells (*n* > 4). **m** dMyc staining in follicle cell mutant clones for *yki*^*B5*^*.***o** dMyc staining in wing disc mutant clones for *Dlg5*^*ex13*^*.* For all pictures scale bar is 10 μm. (** *p*-value < 0.01, **** p *p*-value < 0.0001)

We aimed to define how Dgl5 modulates growth. It has been reported that Dlg5 modulated hippo pathway in the wing. However, this pathway is usually described as controlling of cell proliferation rather than cell growth. The Hippo signaling pathway regulates cell proliferation by inactivating Yorkie (Yki), the Drosophila Homolog of YAP. We therefore induced *yki* RNAi, and, as expected, its loss of function markedly reduces wing size and the estimated number of cells in the whole wing (Fig. 3a-d). Importantly, we also noticed a reduction of cell size in the same range than what has been observed with the loss of *Dlg5* (Fig. 3e). Thus the hippo pathway also modulates cell size and could therefore explain Dlg5 contribution in this tissue. Yki is known to be required for normal follicle cell proliferation [22]*.* We induced null mutant clones and checked for cell size defects at stages 9–10. Comparing the cell surface indicates a moderate but significant effect of *yki* (Fig. 3f-g). Together, these data clearly establish a role for *yki* in the control of cell size. However, this defect appears not stronger than the one induced by the loss of *Dlg5* by RNAi (Fig. 1b). Moreover, inhibition of hippo pathway has not been described as affecting systemic growth, leading to the hypothesis that Dlg5 may modulate growth by at least another means. Looking for other potential growth actors affected by the loss of Dlg5, we noticed that this gene was picked-up in a RNAi screen for TORC1 pathway regulators in S2 cells as affecting the level of S6K protein [17]. However, S6K level was unchanged in follicular cells or wing disc mutant cells for *Dlg5*^*ex13*^ (Fig. 3h,l). Moreover, phosphorylation level of S6 were not affected in *Dlg5* mutant follicle cells, indicating that Dlg5 does not modulate S6K activity and more generally the Tor pathway in this tissue (Fig. 3i,l). We also, checked Insulin/PI3K pathway activity, which when affected, give similar defect both at the cellular and the systemic levels, but we did not observe any alteration of Phospho-Akt in *Dlg5* mutant cells (Fig. 3j,l). Thus, how Dlg5 influences growth in these cells remains to determine. Nonetheless, we noticed a reduction of Myc expression in *Dlg5* mutant follicle cells, suggesting that it is required for the efficient signaling of one of the multiple pathways controlling Myc levels (Fig. 3k,l) [23]. Importantly, Myc levels were never affected in mutant follicle cells for *yki*, demonstrating that Dlg5 effect is independent of Hippo pathway (Fig. 3m). However, similar reduction was not detected in wing disc *Dlg5* mutant cells (Fig. 3n), indicating that Myc regulation cannot account for Dlg5 effect on growth in all tissues.

### Dlg5 is required for the localization of apical polarity determinants

**Fig. 4:**
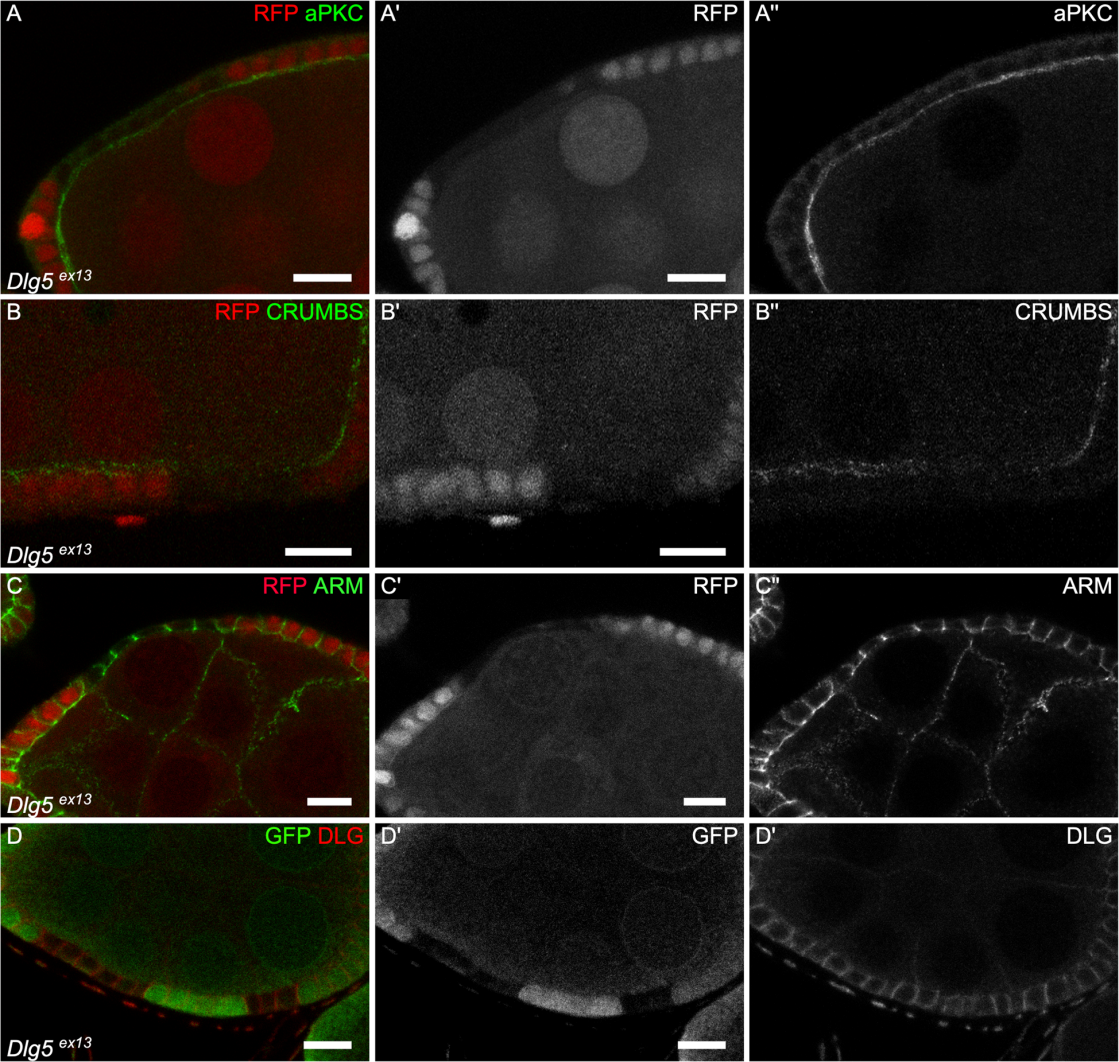
Dlg5 moderately impacts epithelial polarity in follicle cells. **a-d** follicle cell mutant clones for *Dlg5*^*ex13*^ and stained for **a**) aPKC, **b**) Crb, **c**) Arm and **d**) Dlg. For all pictures scale bar is 10 μm

The fact that *Dlg5* regulates growth both in mammals and in fly prompted us to check for an epithelial polarity phenotype, since such a defect has also been observed in *Dlg5* mutant mouse. These defects have been detected for instance in the kidney or the lung, where a partial mislocalization of the apical determinant aPKC, a key component of the apical PAR complex, has been observed [10, 11]. Moreover, knock-down of Dlg5 in follicle cells also give similar phenotypes [13]. In follicular cells mutant for null mutant *Dlg5*^*Ex13*^ we saw a semi-penetrant reduction of the apical level of aPKC, the apical domain of these cells being inwards, at the contact with the germline (12/19 clones) (Fig. 4a). The level of the apical determinant Crumbs (Crb) is also reduced (Fig. 4b). However, this apical reduction of apical determinants was never associated with an extension of lateral markers, such as Coracle (Cora), to the apical domain or to a mispositioning of the adherens junctions. Nonetheless, we pointed out that Cora was often upregulated in the mutant cells (21/33 clones) (Fig. 1c,d), a defect never observed with another septate junction marker such as Dlg (*n* = 9) (Fig. 4d). Moreover, we never spotted multi-layers or round mutant cells. Also, mutant cells tend to flatten, a defect more often observed in young follicles (Fig. 4c). Thus, although it is not sufficient to induce a complete loss of cell polarity in this tissue, *Dlg5* null mutation can affect apical polarity determinants and cell morphology.

**Fig. 5:**
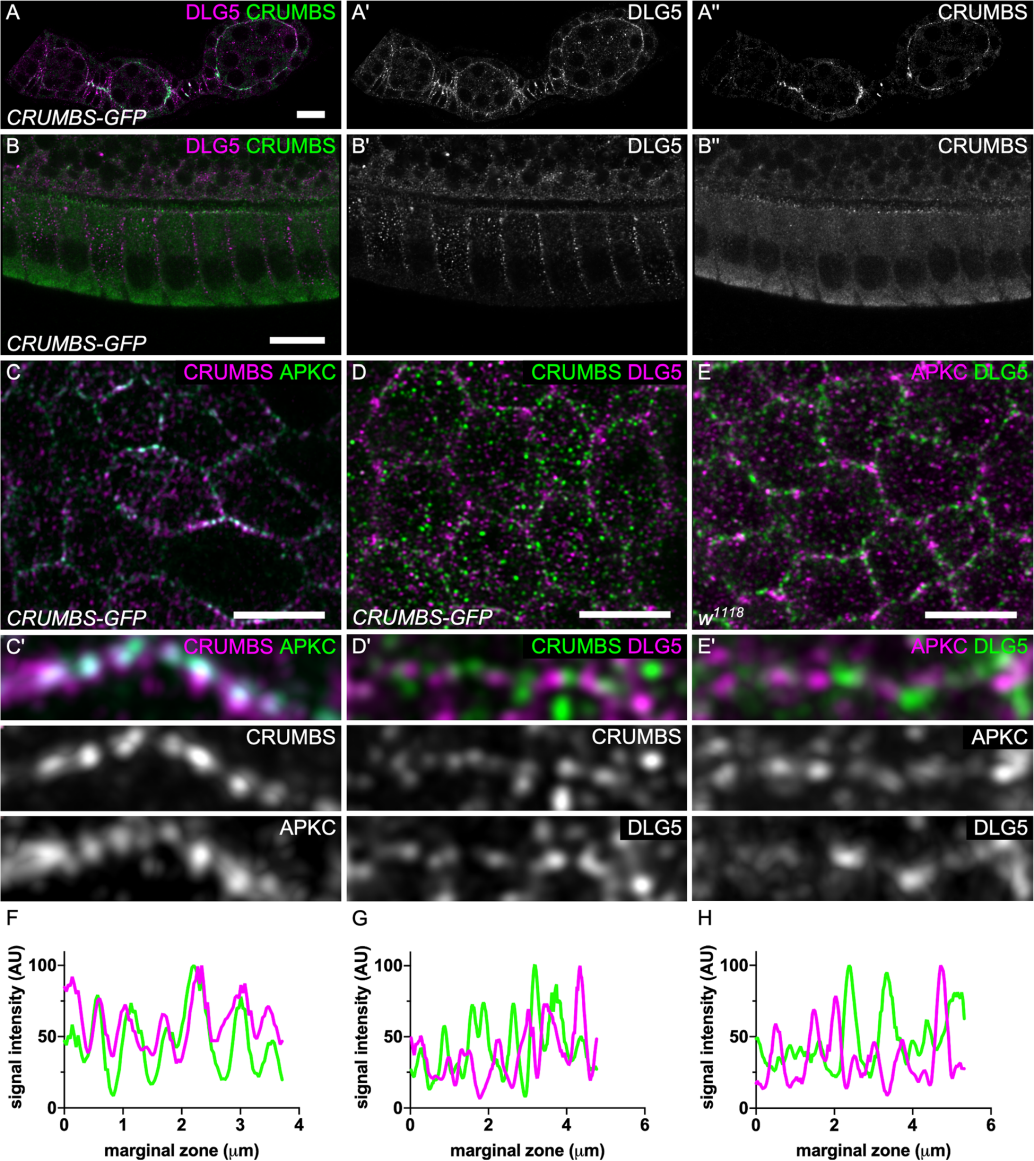
Dlg5 is localized apically but is not colocalized with aPKC and Crb. **a-b** Dlg5 immunostaining on Crb-GFP knock-in follicles on **a**) early stages **b**) stage 10A follicle cells (**c-d**) Airyscan images of the apical plan of Crb-GFP knock-in follicle cells stained for **c**) aPKC D) Dlg5. **e** Airyscan images of the apical plan of WT follicle cells stained for aPKC and Dlg5. **c’d’e**) Zoom-in of the marginal zone of **c**, **d**, **e**. **f**, **g**, **h**) fluorescence intensity profile to the marginal zone shown in **c′, d’** and **e’**. For all pictures scale bar is 10 μm

**Fig. 6:**
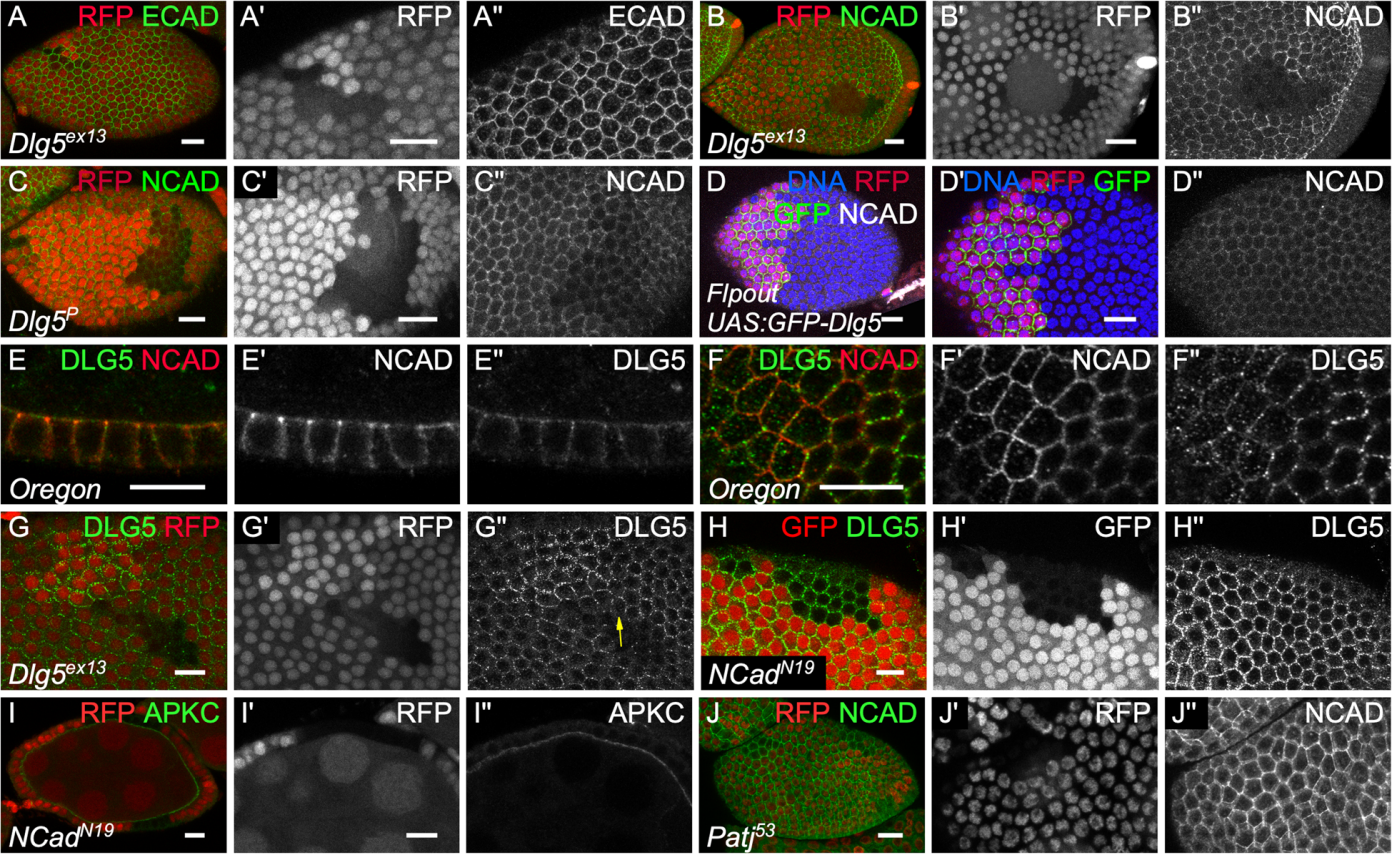
Dlg5 is essential for N-cadherin independently of its effect on polarity. **a, b c** mutant follicle cell clones, marked by the absence of RFP, for (**a**, **b**) *Dlg5*^*EX13*^**c**) *Dlg5*^*KG00748*^ and stained for **a**)E-Cad and **b**)**c**) N-Cad. **d** flip-out clone overexpressing GFP-Dlg5 and stained for N Cad. **e-f** staining on WT follicles of N-cad and Dlg5 . **f** all the cells are not exactly in the same plane and Ncad is enriched more apically than Dlg5. **g***Dlg5*^*EX1*^ mutant follicle cell clones, marked by the absence of RFP and stained for Dlg5. Note the absence of Dlg5 in WT cells at the contact of *Dlg5* mutant cells (arrow). **h, i***Ncad*^*N19*^ mutant follicle cell clones, marked by the absence of RFP and stained for **h**) Dlg5 and **i**) aPKC. **j**) *Patj*^*53*^ mutant follicle cell clones, marked by the absence of RFP and stained for NCad. For all pictures scale bar is 10 μm

To characterize endogenous Dlg5 localization we generated an antibody against the third and fourth PDZ domains. In follicle cells, Dlg5 antibody reveals a dotty pattern, similarly to what has been described in mammals (Fig. 5) [10]. This signal is specific because the antibody gives no signal in Dlg5 mutant follicle cells (Fig. 6g). These dots were observed both inside the cell and at the cell cortex. Moreover, Dlg5 pattern was dynamic depending on the stages. During early stages (2–8) Dlg5 localizes at apical and lateral membranes at stage 1, and appears therefore in apical sooner than Crb (Fig. 5a). Then the apical localization progressively decreases, and is barely detectable at stage 9 (Fig. 2b). Consequently, at later stages this cortical localization is restricted to the lateral domain. Because Dlg5 is present apically as key apical determinants such as aPKC and Crb and affect their localization, we compared their localization in the apical plain of the follicle cells with higher resolution using Airyscan. We observed that aPKC and Crb are usually colocalized, especially at the marginal zone, an area of cell–cell contact apical to the adherens junctions (Fig. 5c,f). This observation comes as confirmation that these proteins cooperate to define the apical domain [24]. In contrast, no evident colocalization is observed between Dlg5 and those two proteins and their localization even tend to be exclusive in the marginal zone, indicating that Dlg5 is not stably associated with these apical determinants (Fig. 5d,e,g,h).

### Dlg5 is required for N-cadherin localization independently of its effect on cell polarity

Looking at the Adherens junction, we found that E-Cad level was not affected (*n* = 18) (Fig. 6a). Follicle cells also expressed N-Cad, which is integrated in adherens junction, and Dlg5 has been functionally and molecularly linked to this cadherin in mammals [10, 12, 25]. We therefore looked at N-Cad and spotted an extremely strong and fully penetrant reduction in *Dlg5* mutant cells (*n* > 20) (Fig. 6b). This effect correlates with the strength of Dlg5 loss of function because the reduction is weaker in hypomorphic conditions (Fig. 6c). However, Dlg5 overexpression does not increase N-Cad levels at cell contacts and has no visible impact on cell size (Fig. 6d). Thus, *Dlg5* loss of function has a dramatic effect on N-Cad, but not on E-Cad, in the same cell type and at the same developmental stages. It indicates therefore a very specific effect on N-Cad membrane delivery or stability.

We therefore looked at a potential colocalization between N-Cad and Dlg5. Both are present mainly at the cell periphery. However, at the adherens junction plain of the cells Dlg5 is mainly found as medioapical dots whereas N-Cad surrounds the cells (Fig. 6f). Just above, less N-cad is observed whereas Dlg5 becomes more enriched at the cortex. As a result, only a weak colocalization between the two proteins is observed with N-Cad being globally more apical than Dlg5 (Fig. 6f). Thus, although Dlg5 has a very strong and specific impact on N-Cad, their potential association is likely transitory. We also noticed that when we induced *Dlg5* mutant cell clones, Dlg5 disappears from the cell cortex even at the boundary with wild-type cells (Fig. 6g). Classical interpretation for such observation is that Dlg5 is associated with proteins performing homophilic interactions between cells and that it is required for their localization, explaining its absence on the wild-type cell side. However, Dlg5 localization is not affected in N-Cad mutant clones (Fig. 6h). Thus, Dlg5 is probably associated with another protein performing homophilic interactions and that is mainly localized at the lateral domain of the follicle cells.

The loss of Dlg5 affects both apical and adherens junction proteins. Since there is important cross-talks between the protein complexes acting at these two sites, we wondered whether those two defects were linked [26–28]. First, the loss of N-Cad had no effect on aPKC apical level, in agreement with the previous proposition that N-Cad and E-Cad are redundant in the follicle cells to maintain adherens junction and epithelial polarity (Fig. 6i) [25]. Thus, Dlg5 impact on N-Cad does not explain the loss of apical proteins. Second, the reduction of apical determinant Crb and aPKC is much less expressive and penetrant than the loss of N-Cad and it is therefore unlikely the cause of such a defect. *Patj* mutation, a component of the Crb complex, leads to a very similar mild effect to *Dlg5* mutation on aPKC and Crb apical levels and on cell morphology [29]. However, *patj* mutant cells show a normal level of N-Cad (Fig. 6j). Thus, the reduction of apical determinants and of N-Cad observed in *Dlg5* mutant cells correspond to two independent functions of the protein.

## Discussion

Altogether, our data show that Dlg5 owns several independent functions in *Drosophila*, suggesting that its scaffold abilities are used in different contexts. Of notice, these functions are somehow opposite to Dlg ones, both on growth and cell polarity, confirming that, despite the same naming, these two MAGUK proteins are unrelated.

First, Dlg5 requirement for growth is really strong, as revealed by the defect of the homozygous mutant larvae and its loss in the wing. It has been recently proposed that Dlg5 is involved in the hippo pathway, via a physical interaction and an inhibition of Hippo kinase by a mechanism that remains to be elucidated [16]. In fly, the main argument for such a regulation is a reduction of the Hippo reporter Expanded-lacZ in a *Dlg5* loss of function in the wing disc. Consistently with this observation, *yki* loss of function induces a reduction of cell size both in the wing and in follicle cells. To our knowledge, this is the first report of a role of this gene in cell size control, which is actually accountable for about 15% of *yki* impact on tissue size. Interestingly, Hippo has been shown to be modulated by TORC1 or InR/PI3K pathways in different contexts [22, 30, 31]. Our results suggest that hippo modulation by these pathways may participate to explain their impact on cell size. Alternatively, these results could suggest that hippo pathway could conversely modulate these pathways. However, several lines of evidence suggest that the regulation of hippo is not sufficient to explain Dlg5 impact on growth. First, in the wing disc, we did not see an effect of *Dlg5* null mutation on Myc, a well-established target of the Hippo pathway in this tissue [32]. Moreover, to the difference of *yki* mutant cells, we did not find evidence that *Dlg5* mutant ones are eliminated by cell competition from the clones [33, 34]. Second, in the follicle cells, the situation looks at the opposite because Myc is modulated by Dlg5 but not by yki. Moreover, the effect of null mutation for *yki* on follicle cell size appears weaker than the one of *Dlg5* hypomorphic conditions induced by RNAi. Finally, we observed a strong requirement of Dlg5 for larval growth, whereas such an effect of *yki* loss of function has never been reported.

*Dlg5* mutant defects are reminiscent of strong mutants for cell growth such as the ones of key components of the TORC1 pathway, with a decrease of cell size and cell proliferation but no induction of apoptosis, even in clonal analysis that can reveal cell competition. However, we did not confirm in vivo the proposed link between Dlg5 and S6K stability or TORC1 activity [17]. Moreover, Myc expression is independent of TORC1 in follicle cells (Vachias, unpublished) whereas it is influenced by this complex in the wing disc [35], a contrary effect to the one of *Dlg5*. Thus, Dlg5 impact on growth is probably independent of TORC1. Similarly, misregulation of InR/PI3K pathway, which impacts cell size and proliferation, has not been observed in *Dlg5* mutant cells. Thus, by which alternative pathway Dlg5 acts on cell growth and tissue size remains to be elucidated.

Our data using null mutants confirm the moderate impact of *Dlg5* on apical-basal polarity observed in fly and mouse [10, 11, 13]. In follicle cells, these defects are really similar to the ones that we previously observed with *Patj* mutation, with both a semi-penetrant loss of apical determinants and a same cell shape defect in follicle cells [29]. This correspondence is in agreement with the proposition that Dlg5 regulates Crb complex [13]. Whereas Crb is essential to maintain follicle cell polarity, this complex is dispensable for the apical basal polarity of the wing cells, as it seems to be the case for Dlg5 because we observed no morphologic defect in this tissue [25, 36]. Interestingly, mouse *Crb3* knock-out leads to cell polarity alteration in the kidney and the lung, which are also the epithelia known to be affected in *Dlg5* mutant mouse, suggesting that the relationship between Dlg5 and Crb complex is conserved throughout evolution [37]. However, high resolution imaging of follicle cell apical domain reveals no colocalization between Dlg5 and Crb, suggesting that the effect of Dlg5 on Crb is indirect or relies on a very transient interaction. Moreover, apical localization of Dlg5 appears sooner than the one of Crb in young follicles suggesting different dynamics for the two proteins. Nonetheless, Crb is also a modulator of the Hippo pathway and it might be interesting in the future to explore the relationship between Dlg5, Crb and Hippo [38–42].

We also observed an extremely strong effect of Dlg5 null mutants on N-Cad localization at the membrane. Dlg5 does not affect E-Cad localization in the same cells and at the same stage, indicating a very specific effect. Although the specificity of the effect on N-Cad versus E-Cad was not established, available data in mammals also denote an impact of Dlg5 on N-Cad delivery associated with a physical interaction between the two proteins [10, 12]. Thus, the specific effect of Dlg5 on N-Cad is likely a conserved feature. E-Cad is usually associated with the acquisition of a stable epithelium architecture whereas N-Cad is more linked to collective cell migration and epithelium-mesenchyme transition [43, 44]. However, despite thousands of articles depicting E-cad versus N-cad expression, the molecular differences underlying these peculiarities are still an open question, relevant for developmental cell biology and cancer. The specific relationship between Dlg5 and N-Cad might provide a nice entry point to understand these differences.

## Conclusions

Together our data show that Dlg5 own several conserved functions that are independent of each other in regulating growth, cell polarity and cell adhesion. Its effect on growth is likely pleiotropic, potentially acting on Hippo pathway but not only. Moreover, we revealed an effect of *yki* on cell size. Finally, this work reveals a differential regulation of E-cadherin and N-cadherin localization.

## Methods

### Molecular biology and antibody production

For transgenesis, Dlg5 coding sequence was amplified by PCR and cloned in phase in pUBi:GFP-Nterm-Gateway-AttB vector [45]. Transgenes were generated at AttP3-B landing site. Antibody were raised in rabbit against a fragment of Dlg5 corresponding to amino acids 1260 to 1600 fused to GST (Eurogentec).

### Genetics

Fly were raised on wheat flour (8%) yeast extract (8%), Agar (1.1%) with antifungal and antibiotics. Dlg5 mutants were generated by imprecise excision of P {SUP-orP}KG00748. Excisions that were lethal when crossed with a deficiency covering Dlg5 locus were analyzed at the molecular level. *Dlg5*^*Ex13*^ contains a 1.5 kb deletion going from upstream the transcription start to 200 bp downstream of the translational start. The detailed genotypes, temperature and heat-shock conditions are given in supplemental Table S1.

### Immunostaining and imaging

Dissection and immunostaining were performed as described previously [46], adding CaCl_2_ 1 mM during fixation, excepted for Crb staining, which requires a specific fixation [25]. Primary antibodies used are DE-Cad (1/100, DHSB #DCAD2) and N-Cad (1/100, DHSB, #DN-Ex), Cora (1/200, DHSB, #C615.16), cleaved DCP-1 (1/1000, Cell Signaling, #9578), S6K (1/2000, [47]), pS6K (1/400, [48], pAKT (1/500, Cell signaling, #4054),, dMyc (1/500, SantaCruz BioTechnology #), Crb (1/50, DHSB, #Cq4), aPKC (1/500, Santa Cruz Biotechnology, #C-20G), Dlg (1/200, DHSB, #4F3), Dlg5 (1/ 500, this study). Images were taken using a Leica SP5 confocal microscope or a Zeiss 800 Airyscan. Wing images were acquired on a Zeiss Axio Scan Z1.

Cell segmentation and size quantification were performed on Fiji. For wing disc clones, total size of the clone and cell number were determined and cell size was inferred from these values. Comparison were performed with twin WT cells. For adult wings, Fijiwings was used to determine wing size and cell density in the same posterior region on all images [49]. Cell size and total cell numbers were inferred from these values. In follicles, all the cells were segmented based on Cora staining using Tissue Analyzer [50] and comparison was realized with WT or heterozygous cells in the vicinity of the mutant (or RNAi) cells. Fluorescent signal was measures in the whole cell surface excepted for pAKT for which only signal at junctions between mutant cells was compare to the signal between WT cells.

All the statistical analyses were performed on Prism using t-test. Figures were assembled with ScientiFig [51].

## Supplementary information

**Additional file 1.**

## Data Availability

New materials (mutants, antibodies) will be shared upon request. The datasets used and/or analysed during the current study available from the corresponding author on reasonable request.

## Abbreviations

Dlg: Discs lage
Dlg5: Discs large 5
Tor: Target or Rapamycin
E-Cad: Epithelial-Cadherin
N-Cad: Neuronal-Cadherin
MAGUK: Membrane Associated Guanylate Kinase
PDZ: Post-synaptic density protein 95 Discs large and Zona occludens 1
aPKC: Atypical protein kinase C
crb: Crumbs
GFP: Green Fluorescent Protein
DCP-1: Drosophila Caspase 1
S6K: S6 kinase
yki: yorkie
Patj: Pals1-Associated Tight Junction
InR: Insulin Receptor
PI3K: Phosphatidylinositol 3-kinase

## References

[1] Flores-Benitez D, Knust E (2016). Dynamics of epithelial cell polarity in Drosophila: how to regulate the regulators?. Curr Opin Cell Biol.

[2] Zhu J, Shang Y, Zhang M (2016). Mechanistic basis of MAGUK-organized complexes in synaptic development and signalling. Nat Rev Neurosci.

[3] Li Y, Wei Z, Yan Y, Wan Q, Du Q, Zhang M (2014). Structure of crumbs tail in complex with the PALS1 PDZ-SH3-GK tandem reveals a highly specific assembly mechanism for the apical crumbs complex. Proc Natl Acad Sci U S A.

[4] Zhu J, Shang Y, Xia C, Wang W, Wen W, Zhang M (2011). Guanylate kinase domains of the MAGUK family scaffold proteins as specific phospho-protein-binding modules. EMBO J.

[5] Beutel O, Maraspini R, Pombo-García K, Martin-Lemaitre C, Honigmann A (2019). Phase Separation of Zonula Occludens Proteins Drives Formation of Tight Junctions. Cell.

[6] Schwayer C, Shamipour S, Pranjic-Ferscha K, Schauer A, Balda M, Tada M, Matter K, Heisenberg CP (2019). Mechanosensation of Tight Junctions Depends on ZO-1 Phase Separation and Flow. Cell.

[7] Abbott LA, Natzle JE (1992). Epithelial polarity and cell separation in the neoplastic l(1)dlg-1 mutant of Drosophila. Mech Dev.

[8] Bilder D, Li M, Perrimon N (2000). Cooperative regulation of cell polarity and growth by Drosophila tumor suppressors. Science.

[9] Woods DF, Hough C, Peel D, Callaini G, Bryant PJ (1996). Dlg protein is required for junction structure, cell polarity, and proliferation control in Drosophila epithelia. J Cell Biol.

[10] Nechiporuk T, Fernandez TE, Vasioukhin V (2007). Failure of epithelial tube maintenance causes hydrocephalus and renal cysts in Dlg5−/− mice. Dev Cell.

[11] Nechiporuk T, Klezovitch O, Nguyen L, Vasioukhin V (2013). Dlg5 maintains apical aPKC and regulates progenitor differentiation during lung morphogenesis. Dev Biol.

[12] Wang SH, Celic I, Choi SY, Riccomagno M, Wang Q, Sun LO, Mitchell SP, Vasioukhin V, Huganir RL, Kolodkin AL (2014). Dlg5 regulates dendritic spine formation and synaptogenesis by controlling subcellular N-cadherin localization. J Neurosci.

[13] Luo J, Wang H, Kang D, Guo X, Wan P, Wang D, Chen J (2016). Dlg5 maintains apical polarity by promoting membrane localization of crumbs during Drosophila oogenesis. Sci Rep.

[14] Aranjuez G, Kudlaty E, Longworth MS, McDonald JA (2012). On the role of PDZ domain-encoding genes in Drosophila border cell migration. G3 (Bethesda).

[15] Luo J, Zhou P, Guo X, Wang D, Chen J (2019). The polarity protein Dlg5 regulates collective cell migration during Drosophila oogenesis. PLoS One.

[16] Kwan J, Sczaniecka A, Heidary Arash E, Nguyen L, Chen CC, Ratkovic S, Klezovitch O, Attisano L, McNeill H, Emili A, Vasioukhin V (2016). DLG5 connects cell polarity and hippo signaling protein networks by linking PAR-1 with MST1/2. Genes Dev.

[17] Lindquist RA, Ottina KA, Wheeler DB, Hsu PP, Thoreen CC, Guertin DA, Ali SM, Sengupta S, Shaul YD, Lamprecht MR, Madden KL, Papallo AR, Jones TR, Sabatini DM, Carpenter AE (2011). Genome-scale RNAi on living-cell microarrays identifies novel regulators of Drosophila melanogaster TORC1-S6K pathway signaling. Genome Res.

[18] Horne-Badovinac S, Bilder D (2005). Mass transit: epithelial morphogenesis in the Drosophila egg chamber. Dev Dyn.

[19] Bastock R, St Johnston D (2008). Drosophila oogenesis. Curr Biol.

[20] Smith BN, Ghazanfari AM, Bohm RA, Welch WP, Zhang B, Masly JP (2015). A Flippase-Mediated GAL80/GAL4 Intersectional Resource for Dissecting Appendage Development in Drosophila. G3 (Bethesda).

[21] Ramanathan SP, Krajnc M, Gibson MC (2019). Cell-Size Pleomorphism Drives Aberrant Clone Dispersal in Proliferating Epithelia. Dev Cell.

[22] Borreguero-Muñoz N, Fletcher GC, Aguilar-Aragon M, Elbediwy A, Vincent-Mistiaen ZI, Thompson BJ (2019). The hippo pathway integrates PI3K-Akt signals with mechanical and polarity cues to control tissue growth. PLoS Biol.

[23] Vincent JP, Fletcher AG, Baena-Lopez LA (2013). Mechanisms and mechanics of cell competition in epithelia. Nat Rev Mol Cell Biol.

[24] Morais-de-Sá E, Mirouse V, St Johnston D (2010). aPKC phosphorylation of bazooka defines the apical/lateral border in Drosophila epithelial cells. Cell.

[25] Tanentzapf G, Smith C, McGlade J, Tepass U (2000). Apical, lateral, and basal polarization cues contribute to the development of the follicular epithelium during Drosophila oogenesis. J Cell Biol.

[26] Coopman P, Djiane A (2016). Adherens junction and E-cadherin complex regulation by epithelial polarity. Cell Mol Life Sci.

[27] St Johnston D, Ahringer J (2010). Cell polarity in eggs and epithelia: parallels and diversity. Cell.

[28] Tepass U (2012). The apical polarity protein network in Drosophila epithelial cells: regulation of polarity, junctions, morphogenesis, cell growth, and survival. Annu Rev Cell Dev Biol.

[29] Pénalva C, Mirouse V (2012). Tissue-specific function of Patj in regulating the crumbs complex and epithelial polarity. Development.

[30] Parker J, Struhl G (2015). Scaling the Drosophila wing: TOR-dependent target gene access by the hippo pathway transducer Yorkie. PLoS Biol.

[31] Straßburger K, Tiebe M, Pinna F, Breuhahn K, Teleman AA (2012). Insulin/IGF signaling drives cell proliferation in part via Yorkie/YAP. Dev Biol.

[32] Neto-Silva RM, de Beco S, Johnston LA (2010). Evidence for a growth-stabilizing regulatory feedback mechanism between Myc and Yorkie, the Drosophila homolog of yap. Dev Cell.

[33] Huang J, Wu S, Barrera J, Matthews K, Pan D (2005). The hippo signaling pathway coordinately regulates cell proliferation and apoptosis by inactivating Yorkie, the Drosophila homolog of YAP. Cell.

[34] Thompson BJ, Cohen SM (2006). The hippo pathway regulates the bantam microRNA to control cell proliferation and apoptosis in Drosophila. Cell.

[35] Parisi F, Riccardo S, Daniel M, Saqcena M, Kundu N, Pession A, Grifoni D, Stocker H, Tabak E, Bellosta P (2011). Drosophila insulin and target of rapamycin (TOR) pathways regulate GSK3 beta activity to control Myc stability and determine Myc expression in vivo. BMC Biol.

[36] Salis P, Payre F, Valenti P, Bazellieres E, Le Bivic A, Mottola G (2017). Crumbs, Moesin and Yurt regulate junctional stability and dynamics for a proper morphogenesis of the Drosophila pupal wing epithelium. Sci Rep.

[37] Whiteman EL, Fan S, Harder JL, Walton KD, Liu CJ, Soofi A, Fogg VC, Hershenson MB, Dressler GR, Deutsch GH, Gumucio DL, Margolis B (2014). Crumbs3 is essential for proper epithelial development and viability. Mol Cell Biol.

[38] Grusche FA, Richardson HE, Harvey KF (2010). Upstream regulation of the hippo size control pathway. Curr Biol.

[39] Grzeschik NA, Parsons LM, Allott ML, Harvey KF, Richardson HE (2010). Lgl, aPKC, and crumbs regulate the Salvador/warts/hippo pathway through two distinct mechanisms. Curr Biol.

[40] Ling C, Zheng Y, Yin F, Yu J, Huang J, Hong Y, Wu S, Pan D (2010). The apical transmembrane protein crumbs functions as a tumor suppressor that regulates hippo signaling by binding to expanded. Proc Natl Acad Sci U S A.

[41] Robinson BS, Huang J, Hong Y, Moberg KH (2010). Crumbs regulates Salvador/warts/hippo signaling in Drosophila via the FERM-domain protein expanded. Curr Biol.

[42] Varelas X, Samavarchi-Tehrani P, Narimatsu M, Weiss A, Cockburn K, Larsen BG, Rossant J, Wrana JL (2010). The crumbs complex couples cell density sensing to hippo-dependent control of the TGF-β-SMAD pathway. Dev Cell.

[43] Pieters T, van Roy F (2014). Role of cell-cell adhesion complexes in embryonic stem cell biology. J Cell Sci.

[44] Loh CY, Chai JY, Tang TF, Wong WF, Sethi G, Shanmugam MK, Chong PP, Looi CY. The E-cadherin and N-cadherin switch in epithelial-to-Mesenchymal transition: signaling, therapeutic implications, and challenges. Cells. 2019;8. 10.3390/cells8101118 PMC6830116 31547193

[45] Couderc JL, Richard G, Vachias C, Mirouse V (2017). Drosophila LKB1 is required for the assembly of the polarized actin structure that allows spermatid individualization. PLoS One.

[46] Vachias C, Fritsch C, Pouchin P, Bardot O, Mirouse V (2014). Tight coordination of growth and differentiation between germline and soma provides robustness for drosophila egg development. Cell Rep.

[47] Hahn K, Miranda M, Francis VA, Vendrell J, Zorzano A, Teleman AA (2010). PP2A regulatory subunit PP2A-B' counteracts S6K phosphorylation. Cell Metab.

[48] Romero-Pozuelo J, Demetriades C, Schroeder P, Teleman AA (2017). CycD/Cdk4 and Discontinuities in Dpp Signaling Activate TORC1 in the Drosophila Wing Disc. Dev Cell.

[49] Dobens AC, Dobens LL (2013). FijiWings: an open source toolkit for semiautomated morphometric analysis of insect wings. G3 (Bethesda).

[50] Aigouy B, Umetsu D, Eaton S (2016). Segmentation and quantitative analysis of epithelial tissues. Methods Mol Biol.

[51] Aigouy B, Mirouse V (2013). ScientiFig: a tool to build publication-ready scientific figures. Nat Methods.

