# Supplementary material for: Multiple functions of the scaffold protein Discs large 5 in the control of growth, cell polarity and cell adhesion in Drosophila melanogaster
Source: BMC Dev Biol. 2020 Jun 18;20:10. doi: 10.1186/s12861-020-00218-0 (PMC7301484; doi:10.1186/s12861-020-00218-0)
Supplement: Supplementary file 1 — Additional file 1. [file 12861_2020_218_MOESM1_ESM.docx]

**Table S1 : detailed genotypes and specific conditions**

**(**HS : one hour of heatshock at 37°C)

| FIGURE | **Genotype** | Conditions |
| --- | --- | --- |
| FIGURE 1 | | |
| A-B | *y,w,HSflp122/+;* *tub:FRT-stop-FRT-gal4, UAS:GFP/ RNAi Dlg5^KK104086^* | 1HS at 37°C, 3 days at 30°C before dissection |
| C | *y, ,HSflp122/+;* *act:FRT-stop-FRT-gal4, UAS:GFP/ RNAi Dlg5^GD16339^* | 1HS at 37°C, 3 days at 30°C before dissection |
| D | *y,w,HS:flp122/+; FRT40A, Ubi:RFP/FRT40A, Dlg5^KG00748^* | 1HS at 37°C, 3 days at 25°C before dissection |
| E | *y,w,HS:flp122/+; FRT40A, Ubi:RFP/FRT40A, Dlg5^Ex13^* | 1HS at 37°C, 5 days at 25°C before dissection |
| F | *y,w,HS:flp122/+; FRT40A, Ubi:RFP/FRT40A, Dlg5^Ex13^ ,Ubi :GFP-Dlg5* | 1HS at 37°C, 5 days at 25°C before dissection |
| FIGURE 2 | | |
| A | *Nubbin :gal4* | 20°C |
| B-D | *Nubbin :gal4, Dlg5^KK104086^* | 20°C |
| C,E-H | *y,w,HS:flp122/+; FRT40A, Ubi:RFP/FRT40A, Dlg5^Ex13^* | 1HS at 37°C, 2 days at 25°C before dissection |
| I | *FRT40A, Dlg5^Ex13^ ou Df(2L)BSC242/CyOGFP* | 70 HOURS AED at 25°C |
| J | *FRT40A, Dlg5^Ex13^* / Df(2L)BSC242 | 70 HOURS AED at 25°C |
| FIGURE 3 | | |
| A | *Nubbin :gal4* | 20°C |
| B | *Nubbin :gal4* / *RNAi yorkie GD (VDRC 40497 CONSTRUCT 11187 )* | 20°C |
| F | *y,w,HS:flp122/+; FRT42D ,Ubi:GFP / FRT42D Yki^B5^* | 1HS at 37°C, 3 days at 25°C before dissection |
| H | *y,w,HS:flp122/+; Ubi:RFP, FRT40A, / Dlg5^Ex13^ FRT40A* | 1HS at 37°C, 5 days at 25°C before dissection |
| I | *y,w,HS:flp122/+; Ubi:GFP , FRT40A, / Dlg5^Ex13^ FRT40A* | 1HS at 37°C, 5 days at 25°C before dissection |
| J | *y,w,HS:flp122/+; Ubi:GFP , FRT40A, / Dlg5^Ex13^ FRT40A* | 1HS at 37°C, 4 days at 25°C before dissection |
| K | *y,w,HS:flp122/+; Ubi:RFP , FRT40A, / Dlg5^Ex13^ FRT40A* | 1HS at 37°C, 5 days at 25°C before dissection |
| M | *y,w,HS:flp122/+; FRT42D ,Ubi:GFP / FRT42D Yki^B5^* | 1HS at 37°C, 3 days at 25°C before dissection |
| N | *y,w,HS:flp122/+; Ubi:RFP , FRT40A, / Dlg5^Ex13^ FRT40A* | 1HS at 37°C, 2 days at 25°C before dissection |
| FIGURE 4 | | |
| A-C | *y,w,HS:flp122/+; Ubi:RFP , FRT40A, / Dlg5^Ex13^ FRT40A* | 1HS at 37°C, 5 days at 25°C before dissection |
| D | *y,w,HS:flp122/+; Ubi:GFP , FRT40A, / Dlg5^Ex13^ FRT40A* | 1HS at 37°C, 5 days at 25°C before dissection |
| FIGURE 5 | | |
| A-D | *CRUMBS-GFP* | 25°C |
| E | *White ^1118^* | 25°C |
| FIGURE 6 | | |
| A-B | *y,w,HS:flp122/+; Ubi:RFP , FRT40A, / Dlg5^Ex13^ , FRT40A* | 1HS at 37°C, 5 days at 25°C before dissection |
| C | *y,w,HS:flp122/+; Ubi:RFP , FRT40A, / Dlg5 ^P^ , FRT40A* | 1HS at 37°C, 5 days at 25°C before dissection |
| D | *y,w,HSflp122/+;* UAS:GFP-DLG5/+ ; Act*:FRT-stop-FRT-gal4, UAS:RFP/+* | 1HS at 37°C, 5 days at 25°C before dissection |
| E, F | *Oregon* | 25°C |
| G | *y,w,HS:flp122/+; Ubi:RFP , FRT40A, / Dlg5^Ex13^ FRT40A* | 1HS at 37°C, 6 days at 25°C before dissection |
| H | *y,w,HS:flp122/+; Ubi:GFP , FRT40A, / NCad^N19^ , FRT40A* | 1HS at 37°C, 4 days at 25°C before dissection |
| I | *y,w,HS:flp122/+; Ubi:RFP , FRT40A, / NCad^N19^ ,FRT40A* | 1HS at 37°C, 4 days at 25°C before dissection |
| J | *y,w,HS:flp122/+; Ubi:RFP, FRT80B, / Patj ^53^,FRT80B* | 1HS at 37°C, 5 days at 25°C before dissection |
